# Analysis of RAD51C germline mutations in high-risk breast and ovarian cancer families and ovarian cancer patients

**DOI:** 10.1186/1897-4287-10-S2-A84

**Published:** 2012-04-12

**Authors:** ER Thompson, SE Boyle, J Johnson, GL Ryland, S Sawyer, DY Choong, G Chenevix-Trench, AH Trainer, GJ Lindeman, G Mitchell, PA James, IG Campbell

**Affiliations:** 1 Peter MacCallum Cancer Centre, East Melbourne, Australia; 2 Queensland Institute of Medical Research, Herston, Australia; 3 The University of Queensland, Brisbane, Australia; 4 Monash University, Clayton, Australia; 5 The Royal Melbourne Hospital, Parkville, Australia; 6 The Walter and Eliza Hall Institute of Medical Research, Parkville, Australia

## 

The recent identification of a biallelic *RAD51C* (*FANCO*) mutation in a family with a Fanconi Anemia-like disorder led to its examination in a large hereditary breast and ovarian cancer case-control candidate study (Meindl, et al., 2010; Vaz, et al., 2010). Meindl *et al*. identified six independent pathogenic monoallelic mutations. Interestingly, these mutations were identified exclusively within 480 families with breast and ovarian cancer (frequency 1.3%) but not among any of the 620 families with breast cancer only. In most subsequent studies, the mutation frequency has been found to be lower than 1.3%, with only three additional truncating mutations being identified in five families among more than 729 ovarian cancer families (with or without breast cancer) (Akbari, et al., 2010; Pang, et al., 2011; Pelttari, et al., 2011; Romero, et al., 2011; Silvestri, et al., 2011; Wong, et al., 2011; Zheng, et al., 2010). Despite the presence of breast cancer in 10 of the 15 *RAD51C* mutation positive families reported to date, analysis of more than 1,373 breast cancer-only families by eight studies collectively has not identified any additional families with truncating *RAD51C* mutations. Therefore, while evidence of a causative role for *RAD51C* in breast and ovarian or ovarian only cancer families (HBOC) is convincing, albeit with low prevalence, its role in breast cancer only (HBC) families remains unclear.

To provide more definitive data on the incidence of *RAD51C* mutations in hereditary breast and ovarian families, we utilised high resolution melt (HRM) analysis to screen for germline mutations in all coding exons of *RAD51C* in index cases from 1,388 non-*BRCA1*, non-*BRCA2* high risk Australian HBC and HBOC families, and 427 controls. In addition, the contribution of *RAD51C* in unselected ovarian cancer was examined through the analysis of germline DNA from an unselected cohort of 267 ovarian cancer patients.

Analysis of 1,053 HBC and 335 HBOC families, and 267 unselected ovarian cancer cases identified 12 novel heterozygous variants in *RAD51C*, three of which were protein truncating, six non-synonymous, one synonymous and two non-coding. Numerous dbSNPs and variants from previous studies were also identified. Two truncating mutations, c.72_73insTGCGG (p.V25CfsX3) and c.397C>T (p.Q133X), were identified amongst the 1,388 familial cases. Consistent with the previously reported deleterious mutations, both variants were identified in families with at least one report of ovarian cancer. A third truncating variant, c.230delG (p.G77VfsX24), was identified in a high grade serous tumour among the 267 unselected ovarian cancer cases. *In silico* analyses predict that four missense variants (including two novel variants) are likely to be pathogenic. Our data also provide support for the designation of the previously reported missense variants p.G264S, and possibly p.A126T, as moderate penetrance alleles.

